# Coronary Artery Aneurysmal Disease and Acute Coronary Syndrome

**DOI:** 10.1177/2324709616640008

**Published:** 2016-03-21

**Authors:** Wesam Ostwani, Holly Fleming, Carlos A. Roldan

**Affiliations:** 1 University of New Mexico, Albuquerque, NM, USA; 2 New Mexico VA Health Care System, Albuquerque, NM, USA

**Keywords:** aneurysms, coronary, dissection, embolization

## Abstract

The patient is a 70-year-old male with no other atherogenic risk factors who presented with an acute coronary syndrome (ACS) of unstable angina subsequently complicated by a non-ST elevation myocardial infarction (NSTEMI). The patient’s presentation posed 3 unique features: (1) cardiac catheterization demonstrated nonobstructive 3-vessel multi-aneurysmal coronary artery disease with sluggish antegrade coronary flow; (2) a nonobstructive aneurysmal dissection flap based on contrast staining of the mid left anterior descending artery, which may have led to in situ nonocclusive thrombosis and distal microvascular embolization; and (3) successful conservative medical therapy of coronary artery aneurysmal disease (CAAD) complicated with ACS. CAAD has an incidence of 1.5% to 4.9% in adults. The most common etiology of CAAD is atherosclerotic coronary artery disease. There are no guidelines for the management of CAAD complicated by ACS, and controversies exist as to whether conservative, catheter-based, or surgical management should be pursued.

The patient is a 70-year-old male with no known coronary artery disease (CAD) and no atherogenic risk factors except for age, who presented with 4 hours of severe substernal chest pain with radiation to the left shoulder. There were no aggravating factors, and the pain was relieved with sublingual nitroglycerin.

Vital signs were notable only for a blood pressure of 160/96 mmHg. Cardiovascular exam was normal and electrocardiogram demonstrated normal sinus rhythm with minor nonspecific ST changes. The first troponin I level was 0.012 ng/mL, and the initial clinical impression was unstable angina. The patient received a loading dose of clopidogrel and was started on aspirin, intravenous heparin, metoprolol, lisinopril, and atorvastatin. The patient’s course was complicated by recurrent resting angina with unchanged electrocardiogram and subsequent troponin I elevation of 4 ng/mL, consistent with NSTEMI.

Cardiac catheterization demonstrated nonobstructive 3-vessel multi-aneurysmal CAD with sluggish antegrade coronary flow (arrowheads in Figure 1A, C, and D), as well as a nonobstructive dissection flap based on ulceration (arrow in Figure 1A) and contrast staining (arrows in Figure 1B) of the mid left anterior descending artery (LAD). The patient was treated medically. Transthoracic echocardiogram was unremarkable. The patient received intravenous heparin for 48 hours and was discharged home on aspirin, clopidogrel, atorvastatin, and isosorbide mononitrate.

**Figure 1.**
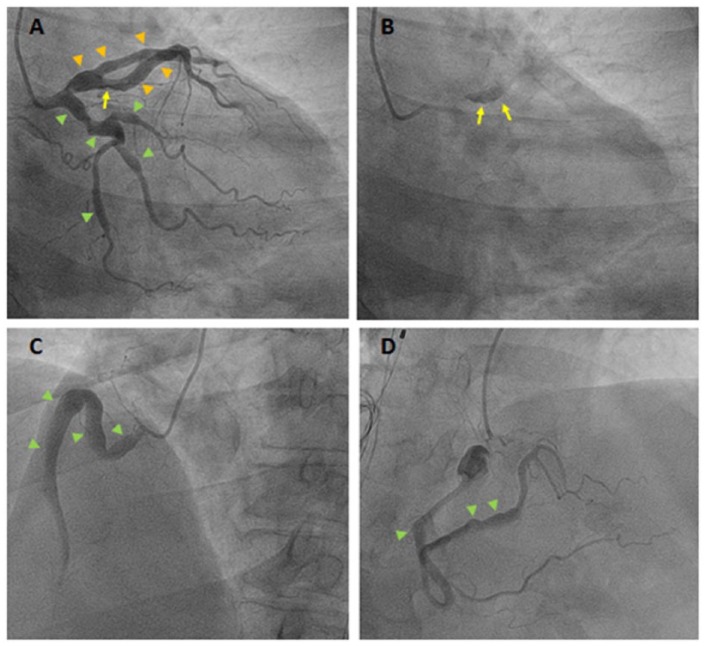
Multivessel coronary artery aneurysmal disease. (A) This angiographic view of the left coronary system demonstrates multiple aneurysmal dilatations of the proximal and mid portions of the LAD and diagonal branch (orange arrowheads). An ulceration (irregular luminal border) is seen in the mid LAD (yellow arrow). Also, note multiple aneurysmal dilatations in the circumflex artery and obtuse marginal branches (green arrowheads). (B) This angiographic view demonstrates contrast staining at the site of the LAD vessel wall ulceration (yellow arrows) suggesting the presence of a nonobstructive intimal flap or dissection. (C, D) Proximal to mid (C) and mid to distal (D) right coronary artery showing multiple areas of aneurysmal dilatation (green arrowheads).

This case presents 3 unique features: (1) 3-vessel involvement with multi-aneurysmal disease. (2) Intimal flap in the aneurysmal mid LAD consistent with aneurysmal dissection. This aneurysmal dissection may have led to in situ thrombosis and distal microvascular embolization, which is another possible cause of acute coronary syndrome (ACS) in coronary artery aneurysmal disease (CAAD). (3) Successful conservative medical therapy of CAAD complicated with NSTEMI.

CAAD is an uncommon clinical finding with an incidence of 1.5% to 4.9% in adults and is defined as coronary artery dilatation that exceeds the diameter of normal adjacent segments or the diameter of the largest coronary artery by a factor of 1.5.^1^ The term aneurysm is used to describe a localized abnormal dilatation of a coronary artery that is either saccular or fusiform in shape, while the term ectasia is used to describe diffuse dilatation.^1^ The term “giant” CAAD is generally reserved for vessel dilation that exceeds the reference vessel diameter by 4 times.^1^

The most common etiology of CAAD is atherosclerotic CAD. However, connective tissue diseases, coronary trauma including iatrogenic injury during percutaneous coronary intervention (PCI), infectious arteritis, Kawasaki syndrome, and congenital CAAD also represent etiologic factors. Congenital CAAD is a possible etiology in our patient based on the absence of atherogenic risk factors. The sequelae of a subclinical infectious or noninfectious arteritis are another consideration in this patient given his multivessel multi-aneurysmal involvement.

CAAD is associated with thrombus formation due to abnormal laminar flow as well as abnormal platelet and endothelial-derived pathophysiologic factors within the aneurysm. Once formed, mural thrombus may potentiate the deposition of additional thrombus within aneurysmal segments. The most likely mechanism for the ACS in our patient was a nonobstructive aneurysmal dissection with in situ nonocclusive thrombosis and distal microvascular embolization given minimal troponin elevation, nonfocal and nonspecific electrocardiogram changes, and normal wall motion on echocardiography.

Presently, there are no guidelines for the management of CAAD complicated by ACS, and controversies exist as to whether conservative, catheter-based, or surgical management should be pursued. According to the reviewed literature, dual antiplatelet therapy (aspirin and clopidogrel or ticagrelor) should be initiated on the identification of CAAD with ACS. Anticoagulation with unfractionated heparin or low-molecular-weight heparin should be added to antiplatelet therapy. If copious thrombus is noted within the aneurysm during angiography, consideration of glycoprotein IIb/IIIa inhibitor infusion for 24 to 48 hours is also recommended.

In cases of CAAD where coronary ischemia persists despite medical optimization, PCI or surgical intervention may be required. PCI of CAAD, particularly in the instance of thrombosis, may pose several technical challenges including distal embolization of thrombus, no-reflow phenomenon, stent malposition, dissection, and rupture. Criteria for surgical revascularization are aneurysms involving the left main coronary artery, multivessel CAAD associated with stenosis, giant CAAD, and CAAD involving bifurcation of significant side-branch vessel.

CAAD is an uncommon clinical finding that poses diagnostic and therapeutic challenges. The case described in this report presents 3 unique features: (1) 3-vessel involvement with multi-aneurysmal disease; (2) aneurysmal dissection with in situ nonocclusive thrombosis and distal microvascular embolization; (3) CAAD complicated with ACS can be successfully managed with conservative medical therapy.
